# Supplementary material for: How two-dimensional bending can extraordinarily stiffen thin sheets
Source: Sci Rep. 2016 Jul 11;6:29627. doi: 10.1038/srep29627 (PMC4939595; doi:10.1038/srep29627)
Supplement: Supplementary Information [file srep29627-s1.pdf]

-SUPPLEMENTARY MATERIALS-

How two-dimensional bending can extraordinarily stiffen thin sheets

V. Pini†, J.J. Ruz†, P. M. Kosaka, O. Malvar, M. Calleja and Javier Tamayo*

IMM-Instituto de Microelectrónica de Madrid (CNM-CSIC), Isaac Newton 8, PTM, E-28760 Tres Cantos, Madrid, Spain

*Corresponding author: jtamayo@imm.cnm.csic.es

S1. Stiffness and resonance frequency of a deformed plate.

We start with a plate with length L, width b and thickness h. The plate is isotropic, homogenous and uniform. The Young's modulus and Poisson's ratio are given by E and ν, respectively. A schematic of the plate and the coordinate system is shown in Fig. 2 of the main text. The length, width and thickness of the undeformed plate are oriented along x-, y- and z-axes, respectively. The origin of the x-axis is at the left free edge of the undeformed plate, and the origins of the y- and z- axis are at the middle of the undeformed plate. We define the normalized coordinates $X = \frac{x}{L}$ and $Y = \frac{y}{b}$. The deformed plate displays an out-of-plane displacement $w_s(X,Y) = \Omega\, L\, n(X,Y)$, where Ω is the maximum static displacement normalized to the plate length and $n(X,Y)$ is the dimensionless function that describes the deformation shape. This initial deformation is referred to as static deformation. We then induce an arbitrarily small deformation, referred to as dynamic deformation, which is characterized by an out of plane displacement $\Delta w(X,Y)$. The relevant dimensionless parameters of the problem are $\beta \equiv \frac{b}{L}$ and $\eta \equiv \frac{h}{L}$. The proposed x and y in-plane displacements induced by the dynamic deformation are given by,

$$u(X,Y,z) = \Omega\, u_0(X,Y) - \frac{\partial \Delta w(X,Y)}{\partial X}\left(\frac{z-\Omega L n(X,Y)}{L}\right) \quad \text{(S1)}$$

$$v(X,Y,z) = \Omega\, v_0(X,Y) - \frac{1}{\beta}\frac{\partial \Delta w(X,Y)}{\partial Y}\left(\frac{z-\Omega L n(X,Y)}{L}\right) \quad \text{(S2)}$$

The Euler-Bernoulli beam bending relations are recovered when the plate is undeformed ($\Omega = 0$). The functions $f(X,Y)$ and $g(X,Y)$ used in the main text are $f(X,Y) = \Omega\, u_0(X,Y)$ and $g(X,Y) = \Omega\, v_0(X,Y)$.

The strains induced by the dynamic deformation are given by,

$$\epsilon_{xx} = \frac{\partial_X u}{L} \qquad \epsilon_{yy} = \frac{\partial_Y v}{\beta L} \qquad \epsilon_{xy} = \frac{1}{2}\left(\frac{\partial_Y u}{\beta L} + \frac{\partial_X v}{L}\right)$$

$$\epsilon_{xz} = \frac{1}{2}\left(\partial_z u + \frac{\partial_X \Delta w}{L}\right) \qquad \epsilon_{yz} = \frac{1}{2}\left(\partial_z u + \frac{\partial_Y \Delta w}{\beta L}\right) \quad \text{(S3)}$$

The stresses are given by[1],

$$\sigma_{xx} = \frac{E}{1-\nu^2}\left(\epsilon_{xx} + \nu\epsilon_{yy}\right) \qquad \sigma_{yy} = \frac{E}{1-\nu^2}\left(\epsilon_{yy} + \nu\epsilon_{xx}\right) \qquad \sigma_{zz} = 0$$

$$\sigma_{xy} = \frac{E}{1+\nu}\epsilon_{xy} \qquad \sigma_{xz} = \frac{E}{1+\nu}\epsilon_{xz} \qquad \sigma_{yz} = \frac{E}{1+\nu}\epsilon_{yz} \quad \text{(S4)}$$

The potential energy is given by,

$$U = \frac{1}{2} L_x b_y \int_0^{\frac{L_x}{L}} dX \int_{-\frac{1}{2}\frac{b_y}{b}}^{\frac{1}{2}\frac{b_y}{b}} dY \int_{w_s(X,Y)-\frac{1}{2}h_z}^{w_s(X,Y)+\frac{1}{2}h_z} \big(\sigma_{xx}\epsilon_{xx} + \sigma_{yy}\epsilon_{yy} + 2\sigma_{xy}\epsilon_{xy} + 2\sigma_{xz}\epsilon_{xz} + 2\sigma_{xz}\epsilon_{xz}\big) dz \quad \text{(S5)}$$

The limits of integration in Eq. (S5) are given by projections of the length, width and thickness of the deformed plate onto the x-, y-, and z- axes, respectively (see Fig. 3 of the main text). In the limit of small deflections, the projected dimensions are given by,

$$L_x \cong L\left(1 - \frac{1}{2}\Omega^2 \int_0^1 \partial_X n^2 dX\right)$$

$$b_y \cong \beta L\left(1 - \frac{1}{2}\left(\frac{\Omega}{\beta}\right)^2 \int_{-\frac{1}{2}}^{\frac{1}{2}} \partial_Y n^2 dY\right)$$

$$h_z = h\left(1 + \frac{1}{2}\Omega^2 \left(\partial_X n^2 + \frac{\partial_Y n^2}{\beta^2}\right)\right) \quad \text{(S6)}$$

We calculate the Euler-Lagrange differential equations obeyed by the unknown functions u_0 and v_0 for the potential energy density integrated along the projected thickness of the plate,

$$\partial_{XX}u_0 + \partial_X(\partial_X n\, \partial_X \Delta w) + \frac{1+\nu}{2\beta}\partial_{XY}v_0 + \frac{1}{2\beta^2}\{(1-\nu)(\partial_{YY}u_0 + \partial_X n\, \partial_{YY}\Delta w + \partial_{YY} n\, \partial_x \Delta w) + (1+\nu)\partial_X(\partial_Y n\, \partial_Y \Delta w)\} = 0$$

$$\partial_{XX}v_0 + \frac{2}{\beta^3(1-\nu)}\{\partial_Y(\partial_Y n\, \partial_Y \Delta w) + \beta\partial_{YY}v_0 + \beta^2[(1+\nu)\partial_Y(\partial_X n\, \partial_X \Delta w) + \partial_{XY}u_0 + (1-\nu)(\partial_{XX} n\, \partial_Y \Delta w + \partial_Y n\, \partial_{XX}\Delta w)]\} = 0 \quad \text{(S7)}$$

Functions $u_0(X,Y)$ and $v_0(X,Y)$ are involved in coupled differential equations that cannot be analytically solved. To obtain an analytical solution to the problem we expand u_0, Δw and n as power series in Y up to the second-order, and the function v_0 up to the third-order term,

$$u_0(X,Y) = u_{00}(X) + \frac{1}{2}u_{02}(X)(\beta Y)^2$$

$$v_0(X,Y) = \left(v_{00}(X) + \frac{1}{2}v_{02}(X)(\beta Y)^2\right)\beta Y$$

$$n(X,Y) = n_0(X) + \frac{1}{2}n_2(X)(\beta Y)^2$$

$$\Delta w(X,Y) = \Delta w_0(X) + \frac{1}{2}\Delta w_2(X)(\beta Y)^2 \quad \text{(S8)}$$

Notice that we assume in Eqns. (S8) that the plate deformation is symmetric in the y- axis. The Euler-Lagrange equations provide four coupled differential equations for the unknown functions $u_{00}(X)$, $v_{00}(X)$, $u_{02}(X)$ and $v_{02}(X)$. An analytical solution can be obtained by neglecting terms $\sim O(\beta^4)$,

$$u_{00}(X) = -\int_0^X \left\{n_0{}'(P)\Delta w_0{}'(P) + \frac{\beta^2}{24}\left(n_2(P)\Delta w_0{}''(P) + n_0''(P)\Delta w_2(P)\right)\right\} dP \quad \text{(S9)}$$

$$u_{02}(X) = -n_2'\Delta w_0' - n_0'\Delta w_2'' - \frac{\beta^2}{120}\{2\nu(2+\nu)(n_0''\Delta w_2' + n_2'\Delta w_0'') - \Delta w_2(3n_2' + 2(2+\nu)\, n_0''') - n_2(3\Delta w_2' + 2(2+\nu)\Delta w_0''')\} \tag{S10}$$

$$v_{00}(X) = -\frac{\nu\beta^2}{24}(n_2\Delta w_0'' + n_0''\Delta w_2) \tag{S11}$$

$$v_{02}(X) = \frac{1}{3}\big(\nu\, n_0''\Delta w_2 - n_2(\nu\Delta w_0'' - 2\Delta w_2)\big) + \frac{\beta^2}{10080}\{(56 + 229\nu - 117\nu^2)(n_2\Delta w_0'''' + n_0''\Delta w_2'' + +n_0''''\Delta w_2 + 2n_0'''\Delta w_2' + 2n_2'\Delta w_0''') + 3(39 - 11\nu)n_2''\Delta w_2 + 39(6 - 19\nu)n_2'\Delta w_2'\} \tag{S12}$$

We substitute Eqns. (S9)-(S12) into the potential energy of the plate Eq. (S5). The resulting potential energy is given by,

$$U = \frac{1}{24}EL\beta\eta\int_0^1\left\{\frac{\eta^2}{1-\nu^2}\left(\Delta w_2{}^2 + \Delta w_0''^2 + 2\nu\Delta w_2\Delta w_0''\right) + \frac{1}{60}\Omega^2\beta^4(\Delta w_2 n_0'' + n_2\Delta w_0'')^2\right\}dX + O(\beta\eta^3\Omega^2) + O(\beta^6\eta\,\Omega^2) \tag{S13}$$

The first summand corresponds to the bending energy (U_b) and the second to the straining energy (U_b). Higher order terms in the bending energy $\sim O(\beta\eta^3\Omega^2)$ and the straining energy $\sim O(\beta^6\eta\,\Omega^2)$ are complex expressions that can be neglected for plates with $\beta < 0.4$. The bending and straining energy can be written in terms of the curvatures of the out-of-plane displacements,

$$U_b = \frac{EL^5\beta\eta^3}{24(1-\nu^2)}\int_0^1\{\Delta\kappa_y(X)^2 + 2\nu\Delta\kappa_y(X)\Delta\kappa_x(X) + \Delta\kappa_x(X)^2\}dX \tag{S14}$$

$$U_s = \frac{EL^7\beta^5\eta}{1440}\int_0^1\big(\kappa_x(X)\Delta\kappa_y(X) + \kappa_y(\mathrm{X})\Delta\kappa_x(X)\big)^2 dX \tag{S15}$$

where $\kappa_x$and $\Delta\kappa_x$ are the static and dynamic x- curvatures at the longitudinal axis of the plate ($Y = 0$); and $\kappa_y$and $\Delta\kappa_y$ are static and dynamic y- curvatures.The relative change of stiffness $\Delta k/k$ and resonance frequency $\Delta f/f$ can be finally evaluated with the following equation,

$$\frac{\Delta k}{k} = 2\frac{\Delta f}{f} = \frac{U_s}{U_b} = \frac{1-\nu^2}{60}\frac{L^2\beta^4}{\eta^2}\frac{\int_0^1\left(\kappa_x\Delta\kappa_y + \kappa_y\Delta\kappa_x\right)^2 dX}{\int_0^1\{\Delta\kappa_y{}^2 + 2\nu\Delta\kappa_y\Delta\kappa_x + \Delta\kappa_x{}^2\}dX} \tag{S16}$$

We analyze the case, in which i) the static curvatures are constants and ii) the sheet is subject to longitudinal bending moment, $\Delta\kappa_y(X) \approx -\nu\Delta\kappa_x(X)$. The relative change of stiffness in this case is independent on how the load is distributed along the plate and reads as,

$$\frac{\Delta k}{k} = \frac{U_s}{U_b} = \frac{L^2}{60}\frac{\beta^4}{\eta^2}\left(\kappa_y - \nu\kappa_x\right)^2 \tag{S17}$$

S2. FEM validation of Free Plate Theory

We compare Eq. (S17) with the results of thorough numerical analysis by the finite element method (FEM) using the commercial software COMSOL Multiphysics. The following boundary conditions were applied:

i) at the plane $Y = 0$, we impose zero-displacement along the Y axis, i.e. $v(X, 0) = 0$
ii) at the plane $X = 0$, we impose zero-displacement along the X axis, i.e. $u(0, Y) = 0$

iii) at the point $(0, 0, 0)$, $w = 0$.

These three boundary conditions restrict the translation and rotation of the plate, but enable free deformation with no clamping restrictions. The parameters swept in the simulations are:

i) The curvatures $\kappa_x$and κ_y were varied from $-4\frac{h}{L^2}$ to $+4\frac{h}{L^2}$.
ii) $\eta = h/L$ was varied from 50 to 300.
iii) $\beta = b/L$ was varied from 0.03 to 0.4.
iv) Poisson's ratio ν was varied from 0 to 0.45.

The total amount of numerical simulations is $\sim 10^5$. For each simulation we applied free tetrahedral meshing and a convergence study was performed by refining the mesh element size until the relative error in the plate eigenfrequency is below 10^{-7}. The average number of degrees of freedom in the simulations was $\sim 10^6$. The numerical simulations were carried out following two sequential steps: i) the plate static deformation is calculated by applying anisotropic differential surface stress in order to independently control the longitudinal and transversal curvatures of the plate[2]; ii) the statically deformed structure is imported and a eigenfrequency analysis is performed. Notice that the plate deformation in a vibration mode fulfils the relation $\Delta\kappa_y(X) \approx -\nu\Delta\kappa_x(X)$ assumed in the derivation of Eq. (S17). The relation between the relative resonance frequency shift and the relative stiffness change is given by $\frac{\Delta k}{k} = 2\frac{\Delta f}{f}$. The numerical analysis shows that the accuracy of Equation (S17) in the main text is always below 10% for free plates in the range of the swept parameters. Fig. S1 plots the comparison between our theory and the FEM simulations of the dimensionless coefficient

$\zeta \equiv \frac{\frac{\Delta f}{f}}{(\kappa L)^2}$ that gives the ratio between the fractional frequency change and the normalized curvature. The comparison is performed for several values of β, η and ν; and for the case $\kappa_x = \kappa_y = \kappa$. Our theory predicts that $\zeta = \frac{(1-\nu)^2 L^2}{120}\frac{\beta^4}{\eta^2}$.

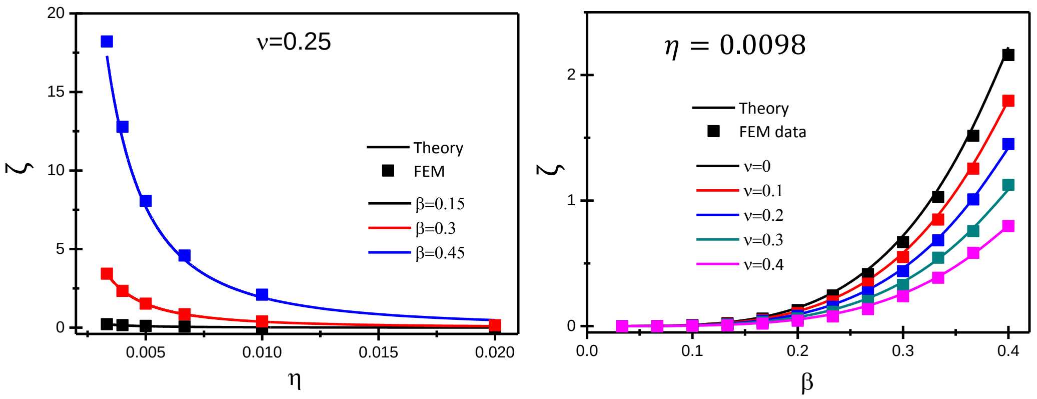

Figure S1. Comparison between the theory (lines) and the FEM data (symbols) of the coefficient ζ for a free plate in the case $\kappa_x = \kappa_y = \kappa$. The values of β, η and ν are specified in the graphs.

S3. Numerical Analysis of Cantilever Plates

The clamping effect in the case of cantilever plates is simulated by restricting the displacement in the three directions at the plane $X = 0$. The same procedure used for the free plate (Sect. S2) was followed. In this case, the FEM data was fitted to,

$$\frac{\Delta k}{k} = \frac{L^2}{60}\frac{\beta^4}{\eta^2} e^{-c\beta}\left(\kappa_y - \nu\kappa_x\right)^2 \quad \text{(S18)}$$

Numerical fittings, performed on about 12k simulations with a coefficient R^2 always higher than 0.999, calculate a constant value of $c = 3.095 \pm 0.001$. Equation (S18) matches the numerical data with an error below 5%.

Fig. S2 plots the comparison between our theory and the FEM simulations of the dimensionless coefficient ζ defined in Sect. S2 for several values of β, η and ν; and for the case $\kappa_x = \kappa_y = \kappa$.

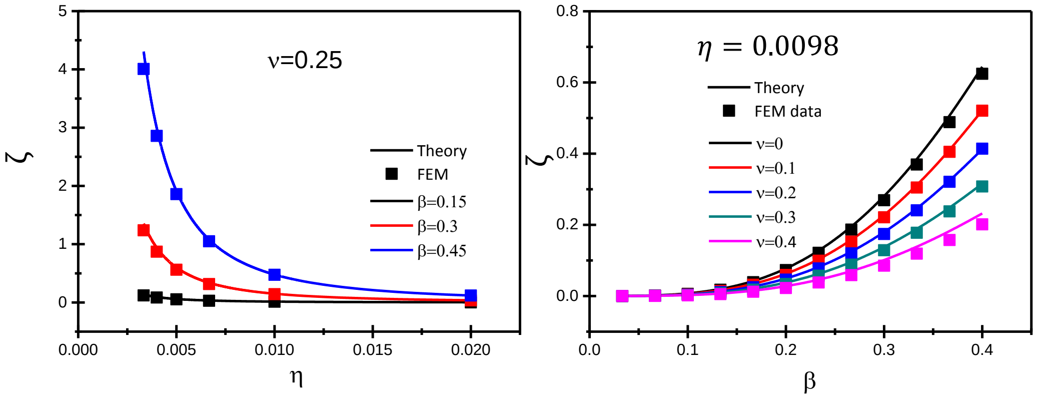

Figure S2. Comparison between the theory (lines) and the FEM data (symbols) of the coefficient ζ for a cantilever plate in the case $\kappa_x = \kappa_y = \kappa$. The values of β, η and ν are specified in the graphs.

S4. Macro Experiment: Stiffness Measurement of a Deformed Aluminum Plate

A plate was fabricated in aluminum with dimensions $29 \times 10 \times 0.0645\ cm^3$. A permanent transversal curvature was achieved pressing the plate against a cylindrical metallic piece. Immediately after, the cantilever is clamped at one of the ends. The transversal curvature was determined by a caliper at the free end, $\kappa_y = 1.15 \pm 0.05\ m^{-1}$. For each case the plate was anchored by one of its edges. An out-of-plane force was manually applied to the free end and then released in order to make it vibrate. The transient oscillation of the plate was imaged by a smartphone camera at a rate of 240 frames per second (IPhone 6s, Apple Inc). A region of the free end was labelled with a red adhesive. The plate oscillation is obtained by tracking the position of the red label in the obtained movie by using MATLAB software. The resonance frequency curve is obtained by calculating the fast Fourier transform of the transient oscillation. The resonance frequency is calculated by fitting the resonance peak to the damped harmonic oscillator equation. The initial longitudinal curvature is obtained by orienting the plate parallel and orthogonal with respect to the gravity force. In the case of the orthogonal orientation, the cantilever plate is subject to gravity, and the longitudinal curvature is given by $\kappa_x(X) \approx \frac{4w_L}{L^2}(X-1)^2$ where w_L is the deflection of the free end[1]. The deflections in our

experiments were $w_L = 2.0 \pm 0.05\, cm$ in the case of zero transversal curvature and $w_L = 1.3 \pm 0.1\, cm$ and $w_L = 0.1 \pm 0.05\, cm$ for the cases transversal curvature downward and upward with respect to gravity, respectively. The difference in deflection between the downward and upward configurations comes from the bending asymmetric effect discussed in the main text. The curvatures of the dynamic deformation are given by $\Delta\kappa_x = \psi''(X)$ and $\Delta\kappa_y = -\nu\, \psi''(X)$ where $\psi(X)$ is the eigenmode shape of the fundamental mode obtained by the Euler-Bernoulli beam theory[3],

$$\psi(X) = \mathrm{Cos}(\alpha X) - \mathrm{Cosh}(\alpha X) + C\big(\mathrm{Sinh}(\alpha X) - \mathrm{Sin}(\alpha X)\big) \quad \text{(S19)}$$

where $C = 0.7340906$ and $\alpha = 1.8751$. Eq. (S16) then transforms into

$$\frac{\Delta f}{f} = \frac{L^2}{120}\frac{\beta^4}{\eta^2} e^{-c\beta} \frac{\int_0^1 \big(\kappa_y - \nu\kappa_x(X)\big)^2 \psi''(X)^2 dX}{\int_0^1 \psi''(X)^2 dX} \quad \text{(S20)}$$

The exponential term accounts for the clamping restriction. The two main sources of error of the theoretical predictions in Fig. 3d of the main text comes from the κ_y uncertainty and from the plate thickness uncertainty, $h = 645 \pm 20\, \mu m$.

REFERENCES

1. Landau, L. D. & Lifshitz, E. Theory of Elasticity, vol. 7. *Course of Theoretical Physics* **3**, 109 (1986).
2. Sader, J. E. Surface stress induced deflections of cantilever plates with applications to the atomic force microscope: Rectangular plates. *Journal of Applied Physics* **89**, 2911-2921 (2001).
3. Tamayo, J., Kosaka, P. M., Ruz, J. J., San Paulo, Á. & Calleja, M. Biosensors based on nanomechanical systems. *Chemical Society Reviews* **42**, 1287-1311 (2013).
